# *Ex vivo* gut culture for studying differentiation and migration of small intestinal epithelial cells

**DOI:** 10.1098/rsob.170256

**Published:** 2018-04-11

**Authors:** Xiaofei Sun, Xing Fu, Min Du, Mei-Jun Zhu

**Affiliations:** 1 School of Food Science, Washington State University, Pullman, WA 99164, USA; 2 Department of Animal Science, Washington State University, Pullman, WA 99164, USA; 3 School of Food Science, University of Idaho, Moscow, ID 83844, USA

**Keywords:** intestinal epithelium, differentiation, AMPK, LGR5, stem cell, migration

## Abstract

Epithelial cultures are commonly used for studying gut health. However, due to the absence of mesenchymal cells and gut structure, epithelial culture systems including recently developed three-dimensional organoid culture cannot accurately represent *in vivo* gut development, which requires intense cross-regulation of the epithelial layer with the underlying mesenchymal tissue. In addition, organoid culture is costly. To overcome this, a new culture system was developed using mouse embryonic small intestine. Cultured intestine showed spontaneous peristalsis, indicating the maintenance of the normal gut physiological structure. During 10 days of *ex vivo* culture, epithelial cells moved along the gut surface and differentiated into different epithelial cell types, including enterocytes, Paneth cells, goblet cells and enteroendocrine cells. We further used the established *ex vivo* system to examine the role of AMP-activated protein kinase (AMPK) on gut epithelial health. Tamoxifen-induced AMPK*α*1 knockout vastly impaired epithelial migration and differentiation of the developing *ex vivo* gut, showing the crucial regulatory function of AMPK *α*1 in intestinal health.

## 1. Introduction

Gut epithelium is one of the largest barriers in the body through forming an integrated single cell layer. The gut epithelial barrier is selectively permeable to nutrients, minerals, water and selected antigens, while protecting against potentially harmful bacteria, viruses and antigenic materials. Neonatal gut has a relatively high permeability, which is required for transferring of maternal immunoglobulins in colostrum and selected antigens, further facilitating the proper development of the gut immune system [1,2]. However, the neonatal gut epithelium is susceptible to pathophysiological perturbations [3–6]. A ‘leaky’ neonatal gut transmits harmful antigens, altering immune system development/maturation and pre-disposing offspring to immunological disorders [7]. The elevated gut permeability is a central predisposing factor to inflammatory bowel diseases, autoimmune and related allergic diseases [8–13]. However, many questions regarding the epithelial differentiation and barrier function, especially during the early gut development, remain to be addressed.

Up to now, studies on epithelial differentiation and migration were mainly using epithelial cell lines and *in vivo* studies. Compared with *in vivo* studies, *in vitro* cell culture is convenient, low cost and time-saving, suitable for using chemical stimulators and inhibitors, and suitable for genetic manipulation without physiological complications. However, cell lines are immortalized cells and their cellular behaviour may differ significantly from normal epithelial cells. The recently developed epithelial organoid culture uses Lgr5 (leucine-rich-repeat-containing G protein-coupled receptor) as a surface marker for isolating gut epithelial stem cells, which further develops into organoid structures using three-dimensional matrigel as a scaffold [14]. This organoid system requires expensive growth factors to facilitate epithelial proliferation and differentiation, and has a relatively low efficiency (1–2%) for epithelial stem cells developing into organoids [15,16]. Furthermore, epithelial organoids do not truly represent *in vivo* epithelial differentiation during gut development due to the lack of underlying mesenchymal tissues that intricately interact with the epithelium to regulate its development [13,17]. Likewise, *in vivo* studies are either costly or impossible to use chemical inhibitors and activators, or RNA interferences, to manipulate epithelial differentiation without inducing physiological complications or other changes. There is a need to establish an *ex vivo* model, which can mimic *in vivo* gut epithelial development but has the convenience and flexibility of *in vitro* cell culture.

Here, we established an *ex vivo* model for studying gut epithelial differentiation and migration using embryonic (E) 13.5-day fetal small intestine. The model can effectively track the differentiation of gut epithelium real-time, which, in combination with the immunohistochemical and histochemical examination of structures, provides a powerful venue for elucidating key mechanisms regulating gut epithelial development.

AMP-activated protein kinase (AMPK) is recognized as a master regulator of energy metabolism [18,19], and was recently recognized for its important regulatory role in myogenesis, adipogenesis, cardiac differentiation, smooth muscle formation and osteogenesis [20–24]. We recently found that AMPK promotes intestinal differentiation *in vitro* and in adult mice [25], but were unable to establish the causal relationship due to the absence of a model for studying AMPK without physiological complications. In this study, the regulatory role of AMPK in regulating embryonic epithelial differentiation and migration was demonstrated using this newly established *ex vivo* gut model.

## 2. Material and methods

### 2.1. Coverslip cleaning, coating and preparation for *ex vivo* gut culture

Coverslips (22 × 22 mm, Thermo Fisher Scientific, Waltham, MA, USA) were dipped into acidic alcohol (0.1% acetic acid in 95% alcohol, V/V), air dried, then submerged into 2% 3-aminoprophytriethoxysilane (^#^440140, Sigma, St Louis, MO, USA) in acetone for 15 min [26]. Coverslips were washed twice in acetone and once in water, and then sterilized by autoclaving. The prepared coverslips could be stored up to one year at room temperature. The centre of each sterile coverslip was coated with 200 µl of 50 µg ml^−1^ fibronectin (^#^610077, BD Biosciences, San Jose, CA, USA). Coverslips were dried under a biosafety cabinet. Before culturing, coverslips were placed into 35 mm cell culture dishes (^#^150318, Thermo Fisher Scientific), and four sterile 4.7 × 8 mm cloning cylinders (^#^C7983, Sigma) were placed onto the fibronectin-coated area on coverslips.

### 2.2. Mice strains

C57BL/6 J mice were purchased from Jackson Laboratory (^#^000664, Bar Harbor, ME, USA). Mice harbouring an Lgr5-EGFP-IRES-creERT2 (Lgr5) allele (^#^008875, Jackson Lab) were crossed with ROSA^mT/mG^ (^#^007576, Jackson Lab) mice to obtain conditional knockout mice (Lgr5^mT/mG^). Epithelial stem cells in *ex vivo* gut from Lgr5^mT/mG^ mice will convert cellular fluorescent protein from red to green signal in Lgr5-positive cells and their derived cells upon induction of 4-hydroxytamoxifen. The double-fluorescent *ex vivo* gut facilitated the tracing of epithelial movement and reorganization of Lgr5-derived cells. Mice with AMPK*α*1-floxed gene (Prkaa1^tm1.1Sjm^/J, ^#^014141, Jackson Lab) were cross-bred with tamoxifen-inducible Cre mice (129-Gt(ROSA)26Sor^tm1(cre/ERT)Nat^/J, ^#^004847, Jackson Lab) as previously described to obtain AMPK*α*1 conditional knockout (KO) mice [27]. In response to 4-hydroxytamoxifen, AMPK*α*1 can be deleted in all tissues of AMPK*α*1 KO mice. Embryonic (E) staging was started from the morning when a copulatory plug was observed and counted as embryonic day 0.5 (E0.5). Pregnant mice at E13.5 were euthanized for *ex vivo* gut preparation.

### 2.3. Embryonic gut isolation and culture

E13.5 embryos were removed aseptically and transferred into ice-cold culture medium (DMEM, Sigma) supplemented with 10% fetal bovine serum (FBS, Invitrogen, Carlsbad, CA, USA) and 1% antibiotic–antimycotic solution (Invitrogen). The small intestine was dissected from embryos according to a procedure described previously with modifications [26]. Briefly, small intestine was cut into 2 mm segments using fine scissors and dissecting needle under a stereomicroscope (Nikon SMZ800, Tokyo, Japan). Then, to each 35 mm dish, a fibronectin-coated coverslip was placed and then 2 ml DMEM supplemented with 20% FBS and 1% antibiotic–antimycotic solution was added. After that, cloning cylinders were placed into dishes, and to each cloning cylinder, 60 µl of medium was added, followed by transferring 2–3 gut sections into the centre of each cloning cylinder. Gut sections were gently pushed down using a dissecting needle so that they could settle on fibronectin-coated coverslips and the dishes containing gut sections were carefully transferred to the 37°C, 5% CO_2_ humidified incubator. The cloning cylinders were carefully removed after 24 h of culture. The gut segments were cultured for 10 days, and the culture medium was changed every 3 days. To determine *Gata4* expression in different segments of small intestine, *ex vivo* guts from embryonic proximal or distal small intestine were cultured and collected at 7 days separately. To evaluate the effects of signalling regulators on the differentiation of *ex vivo* guts, culture medium containing 100 nM LDN-193189 (a BMP signalling inhibitor, Stemolecule, Cambridge, MA), 10 μM DAPT (a Notch signalling inhibitor, Stemolecule) or 100 ng ml^−1^ Wnt 3a and 500 ng ml^−1^ R-spondin1 (Wnt signalling activators, PeproTech, Rocky Hill, NJ) was prepared to treat the guts for 7 days. To induce AMPK knockout, 4-hydroxytamoxifen (250 µM, Sigma) was used during the first three days of culture.

### 2.4. Gut fixation and cryosection

Following the removal of culture medium, *ex vivo* guts were fixed in freshly prepared 4% formaldehyde for 2 h at room temperature and then rinsed with PBS for three times. *Ex vivo* guts were submerged in PBS with 30% sucrose for 1 h at room temperature, which served as a cryoprotectant for gut during freezing. The cultured guts were carefully peeled from coverslips with dissecting blades under a stereo microscope, then vertically embedded into Tissue Tek^R^ OCT compounds (Miles Sakura, Torrance, CA, USA) in embedding moulds (Thermo Fisher Scientific), followed by placement into isopentane (Sigma) pre-cooled by liquid nitrogen for 30 s. The frozen *ex vivo* gut samples were stored at −80°C and sectioned using a cryostat (Leica, Wetzlar, Germany) to 5 µm thickness and mounted directly onto ultra-stick micro slides (Gold Seal, Bedford, MA, USA), then stored at −80°C till staining.

### 2.5. Immunofluorescence staining

Sections were permeabilized in 1% Triton X-100 (Sigma) in PBS at room temperature for 30 min, boiled in 10 mM sodium citrate (pH 6.0, Sigma) for 20 min, incubated with 5% goat serum (Vector, Burlingame, CA, USA) in PBS (pH 7.4) for 1 h to block non-specific binding sites, then incubated with primary antibodies overnight at 4°C. The following antibodies were used: Smooth muscle actin (smooth muscle cells), Thermo Fisher Scientific ^#^MS-113-B, 1 : 50; E-cadherin (epithelial junctions), Life Technologies ^#^33–4000, 1 : 200; Chromogranin A (enteroendocrine cells), Developmental Studies Hybridoma Bank ^#^CPTC-CHGA-1, 1 : 200; Vimentin (fibroblasts), Cell Signaling Technology ^#^5741, 1 : 200; Ki67 (proliferative cells), Cell Signaling Technology ^#^9449, 1 : 200; CDX2 (intestinal transcription factor), Cell Signaling Technology ^#^12306, 1 : 300; β-catenin (adherens junctions), Cell Signaling Technology ^#^9562, 1 : 300; Cytokeratin 8 (columnar epithelium), Developmental Studies Hybridoma Bank ^#^TROMA-I, 1 : 400; Lysozyme (Paneth cells), Thermo Fisher Scientific ^#^PA5-16668, 1 : 800. Then the slides were incubated with respective fluorescent secondary antibodies (goat anti-mouse antibody-Alexa Fluor 555, Cell Signaling Technology ^#^4409; goat anti-mouse antibody-Alexa Fluor 488, Cell Signaling Technology ^#^4408; goat anti-rabbit antibody-Alexa Fluor 555, Cell Signaling Technology ^#^4413; goat anti-rabbit antibody-Alexa Fluor 488, Cell Signaling Technology ^#^4412; goat anti-rat antibody-Alexa Fluor 488, Cell Signaling Technology ^#^4416) diluted in 5% goat serum (1 : 1000) at room temperature for 2 h. Immunofluorescence was imaged using a fluorescence microscope (EVOS FL, Life Technologies). Negative controls were stained with secondary antibody without primary antibody incubation.

### 2.6. Histochemical staining

For Alcian blue staining, tissue sections were stained with 1% Alcian blue solution (pH 2.5, Sigma) for 30 min at room temperature as previously described [28]. Haematoxylin–eosin (H&E) staining was conducted following standard procedures [29]. The images were taken using an inverted microscope (AMG EVOS, Life Technologies).

### 2.7. RT-qPCR

Total RNA was extracted from 10 *ex vivo* gut samples for each group using TRIzol (Sigma). Five hundred nanograms of cDNA was synthesized using a Reverse Transcription kit (Bio-Rad, Hercules, CA, USA). qPCRs for 5 ng cDNA per reaction were performed on a CFX RT-PCR detection system (Bio-Rad) using SYBR Green (Bio-Rad). The primers were designed to cross two exons to prevent amplification of genomic DNA, and primer sequences are shown in the electronic supplementary material, table S1. Five nanograms of cDNAs per reaction from adult mouse intestine and fat tissue were used as positive control (PC) and negative control (NC), respectively. 18S rRNA was used as an internal control.

### 2.8. TUNEL staining

*In situ* apoptosis was determined using TACS TdT-DAB detection kit (Trevigen, Gaithersburg, MD, USA) per the manufacturer's instructions. Briefly, *ex vivo* gut sections were treated with Cytopore and blocked with 3% H_2_O_2_. DNA fragments were labelled with biotin and HRP-conjugated streptavidin, then incubated with DAB substrates. The counterstaining was performed with 1% Methyl Green. The images were taken using Leica DM2000 LED light microscope (Wetzlar, Germany).

### 2.9. Statistical analysis

*Ex vivo* gut section obtained from each fetus was considered as an experimental unit. Statistical analysis was performed using Prism 6 (GraphPad Software, Inc., La Jolla, CA, USA). Data were presented as means ± standard error of the means (s.e.m.). Differences between means were determined using two-tailed *t*-test followed by Duncan's multiple test when appropriate. All experiments were replicated in at least three independent experiments. *p* ≤ 0.05 was considered to be statistically significant.

## 3. Results

### 3.1. Establishment and functionality of *ex vivo* gut

**Figure 1.**
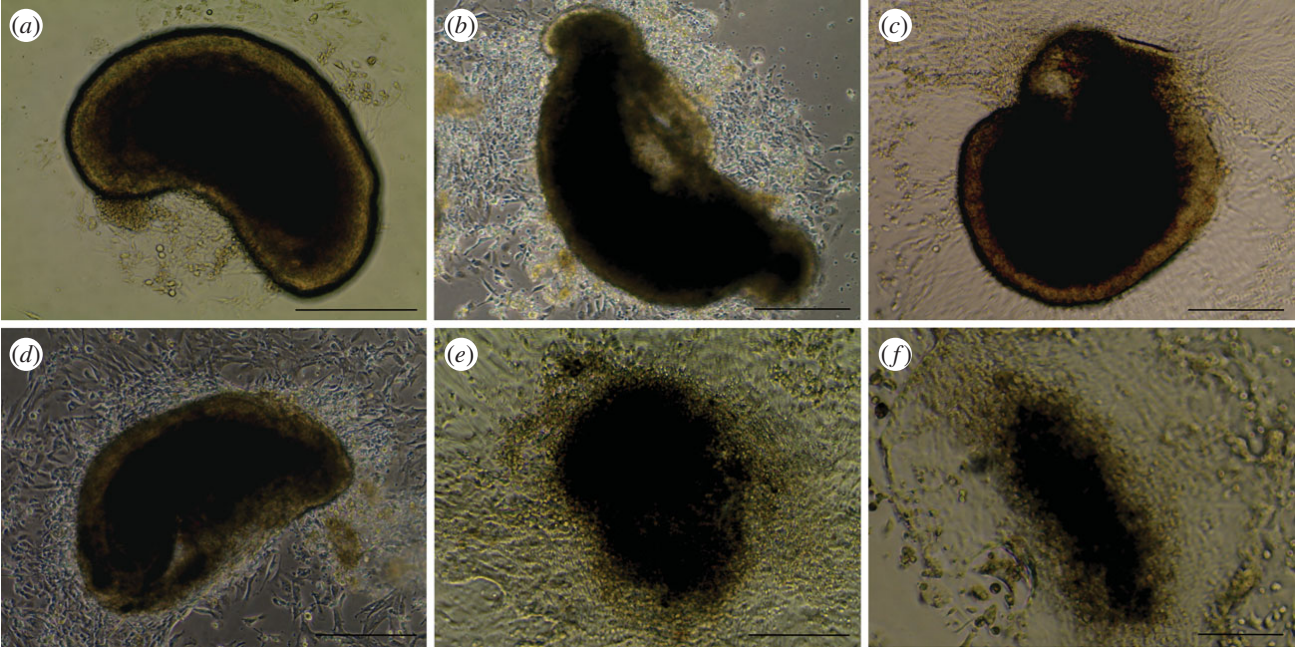
Inverted phase contrast images of *ex vivo* guts. Small intestine from E13.5 embryos were isolated, dissected and cultured to form *ex vivo* guts at 3 days (*a*), 4 days (*b*), 5 days (*c*), 6 days (*d*), 7 days (*e*) and 10 days (*f*). Scale bar is 250 µm.

**Figure 2.**
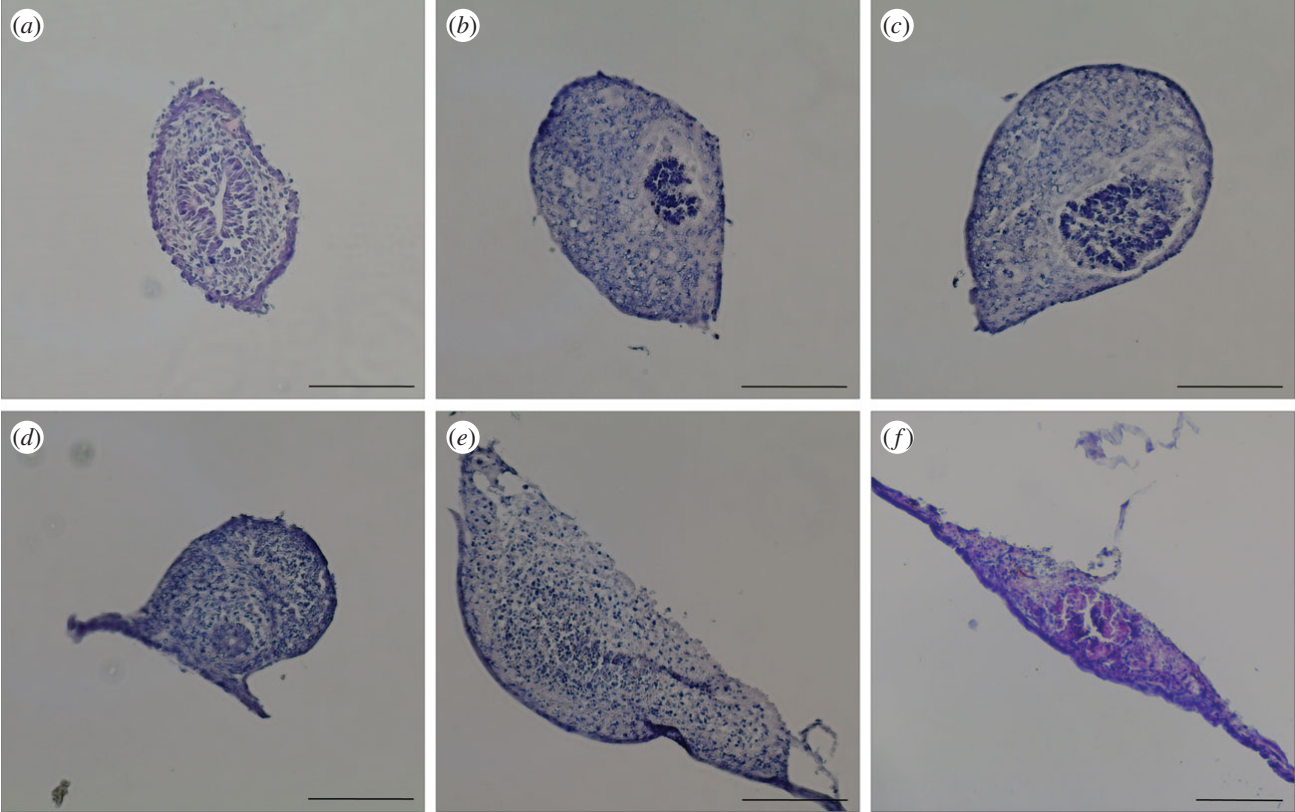
Haematoxylin and eosin staining images of *ex vivo* gut cross-sections. *Ex vivo* guts were collected at day 0 (*a*), and after 3 days (*b*), 4 days (*c*), 5 days (*d*), 7 days (*e*) and 10 days (*f*) of culture, fixed in 4% paraformaldehyde and embedded in OCT, cut into 5 µm section and then stained by haematoxylin and eosin. Scale bar is 125 µm.

The morphology of *ex vivo* gut was examined over 10 days. Gut sections adhered to coverslips within 24 h, and gradually expanded and spread out over time (figure 1). Starting day 3, guts demonstrated autonomous contraction with a frequency of about 15 times per minute (electronic supplementary material, movie S1). To examine the overall reorganization of gut structure over time, guts were cross-sectioned and stained with H&E (figure 2). The gut lumen progressively moved closer to the bottom during the initial 5 days of culture (figure 2*a*–*d*), and guts gradually flattened out from a tubular structure to a spindle-shaped structure over 10 days (figure 2). To determine cell viability during the 10-day culture, Trypan blue staining was performed (electronic supplementary material, figure S1). Sporadic dead cells were found in ‘spread’ area at 7 or 10 days of culture (electronic supplementary material, figure S1*c*–*f*). Addition of Wnt3a and R-spondin1 could extend the culture viability of *ex vivo* guts to 3 weeks. To further examine the functionality of epithelial cells, the activity of alkaline phosphatase produced by epithelial absorptive cells was tested (electronic supplementary material, figure S1 g). Compared with day 0, the activity increased greatly at 7 days, indicating *ex vivo* gut remained functional even though cell death slightly occurred.

### 3.2. Epithelial cell differentiation in cultured gut

**Figure 3.**
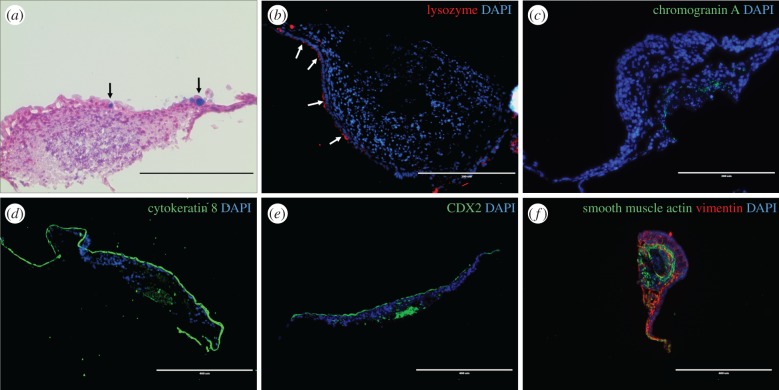
Localization and distribution of cell differentiation markers in *ex vivo* guts 7 days post-incubation. *Ex vivo* guts were cultured for 7 days, fixed and embedded in OCT. The cryosections were examined by Alcian blue staining (*a*) and immunofluorescence staining (*b*–*f*). (*a*) Goblet cells locate on the surface layer of *ex vivo* guts shown by Alcian blue staining (black arrows); (*b*) Paneth cells (white arrows, lysozyme); (*c*) enteroendocrine cells (chromogranin A staining); (*d*) columnar epithelium (cytokeratin 8); (*e*) intestinal transcript factor CDX2; (*f*) fibroblasts (vimentin), smooth muscle cells (smooth muscle actin) and myofibroblasts (co-immunostaining of smooth muscle actin and vimentin). DAPI was used as nuclei stain. Scale bar is 200 µm in (*a*–*c*) and 400 µm in (*d*–*f*).

To characterize the differentiation and distribution of intestinal epithelial cells in cultured guts, *ex vivo* guts at 7 days were stained for the respective differentiated cells. The presence of goblet cells and Paneth cells were visible on the surface of gut section (figure 3*a*,*b*). Enteroendocrine cells were identified in the centre of gut lumen, suggested by chromogranin A staining (figure 3*c*). Absorptive cells, indicated by the columnar epithelial marker cytokeratin 8, formed a tight layer on the outside surface of cultured guts, with a small number concentrated in the centre of the original gut lumen (figure 3*d*). CDX2, the key epithelial transcriptional factor, had a similar distribution to absorptive cells, which might be due to its crucial role in intestinal differentiation (figure 3*e*). By contrast, the remaining components were composed of mesenchymal cells, which were concentrated under the epithelium, providing scaffolds for epithelial cells (figure 3*f*). Fibroblasts formed the first scaffold layer under epithelium, as indicated by vimentin staining (figure 3*f*). Smooth muscle cells formed the second scaffold layer under fibroblasts based on smooth muscle actin staining (figure 3*f*). Myofibroblasts were developed in the spreading area (gut ‘tail’), as suggested by co-staining of vimentin and smooth muscle actin (figure 3*f*). To further test whether interstitial cells of Cajal contribute to spontaneous peristalsis in *ex vivo* guts [30], mRNA expression of *C-kit* was tested, which was beyond the detection level (electronic supplementary material, figure S3a). Furthermore, the peristalsis was promoted after treatment with a neurotoxin, botulinum (electronic supplementary material, figure S3c). The expression of *Gata4* in *ex vivo* guts at 7 days from embryonic proximal small intestine was higher than the one from distal small intestine, indicating the segment specificity in cultured gut (electronic supplementary material figure S1h). Additionally, Wnt activators, BMP inhibitor and Notch inhibitor could alter the expression of differentiation markers of *ex vivo* gut (electronic supplementary material, figure S4), suggesting these signalling pathways regulate the fates of intestinal stem cells during differentiation.

### 3.3. Migration and polarity of cultured gut

We observed the presence of epithelial layers on the surface of *ex vivo* gut, which were unexpected, because epithelial cells were anticipated to concentrate inside the gut lumen. To address the originality of the surface epithelial layers, *ex vivo* guts isolated from intestinal stem cell-derived fluorescent mice (Lgr5^mT/mG^) were further used for tracing epithelial movement and reorganization.

**Figure 4.**
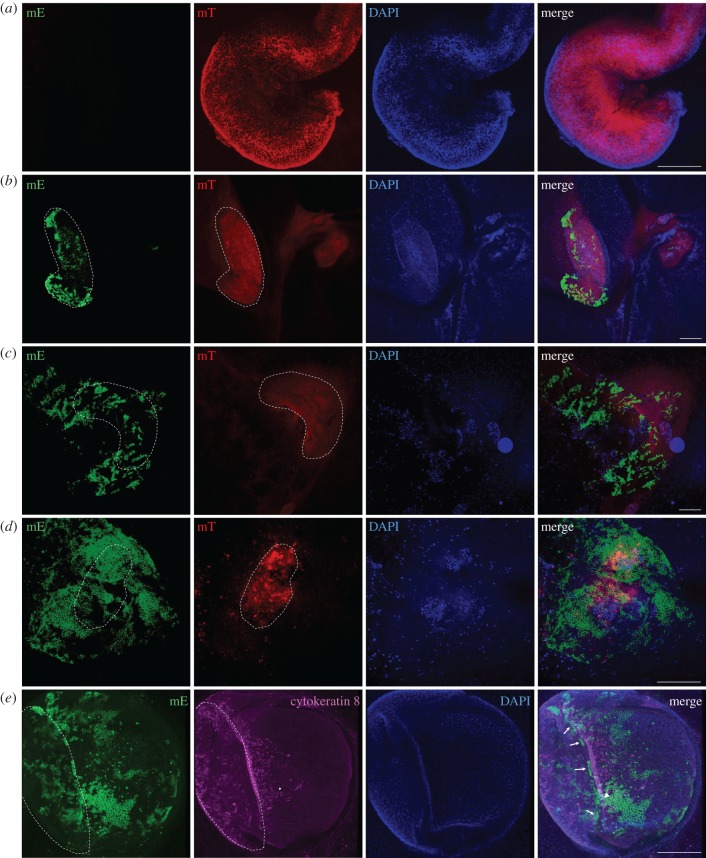
Migration and reorganization of *ex vivo* guts. Lgr5^mT/mG^ *ex vivo* guts were obtained by crossing Lgr5-EGFP-IRES-creERT2 mice with ROSA^mT/mG^ mice. Lgr5^mT/mG^ *ex vivo* guts were induced by 250 µm 4-hydroxytamoxifen in the first 3 days of culture to mark Lgr5-derived cells into green fluorescence. The fluorescent signal at the surface of *ex vivo* guts was observed at day 0 (*a*), and after 3 days (*b*), 6 days (*c*) and 9 days (*d*), by confocal microscope. Columnar epithelium at 7 days is shown by immunostaining for Cytokeratin 8 (*e*). The dashed lines indicate the contour of the original gut body. Arrows point to Lgr5-derived cell clusters. Arrowhead points to cells across the border between original gut body and the cells spreading from the gut body. DAPI was used as a nuclear stain. Scale bar is 200 µm.

**Figure 5.**
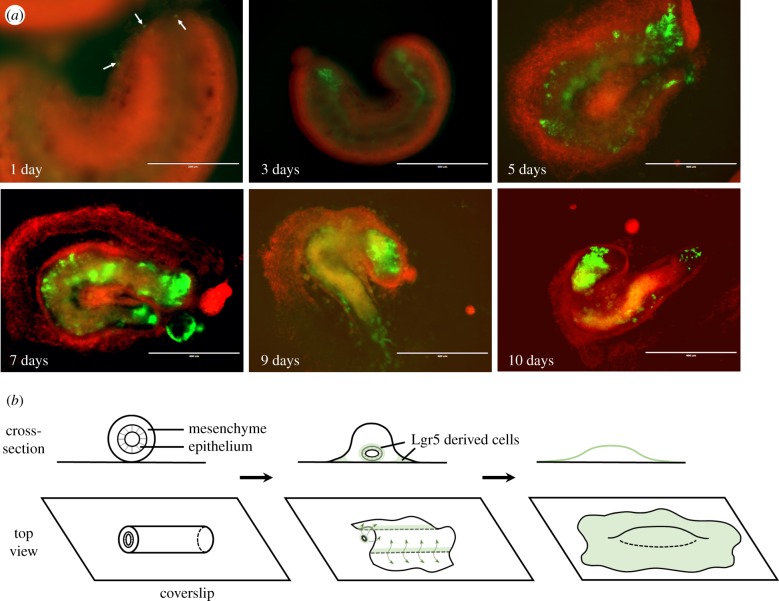
Development of Lgr5 progeny cells and cell signalling in *ex vivo* guts. (*a*) Lgr5^mT/mG^ *ex vivo* guts were obtained by crossing Lgr5-EGFP-IRES-creERT2 mice with ROSA^mT/mG^ mice. Lgr5^mT/mG^ *ex vivo* guts were induced by 250 μm 4-hydroxytamoxifen in the first 3 days of culture to mark Lgr5-derived cells by the presence of green fluorescence. The fluorescent signal under *ex vivo* guts (view from the bottom of Petri dish) was observed at 1, 3, 5, 7, 9 and 10 days by fluorescent inverted microscope. (*b*) Infographic of morphogenesis and migration in *ex vivo* guts. Cross-section view is above and the top view is below.

At day 0, e*x vivo* guts of Lgr5^mT/mG^ showed exclusively red fluorescence (figure 4*a*). During 9 days of culture, residual cells with red fluorescence remained clearly visible (figure 4*a*–*d*). Of note, newly Lgr5-derived epithelial cells with green fluorescence appeared and concentrated at the two ends of gut sections at day 3 (figure 4*b*; electronic supplementary material, movie S2), and further progressively spread along the surface of the gut at day 6 (figure 4*c*; electronic supplementary material, movie S3), leading to almost complete coverage of gut at day 9 (figure 4*d*; electronic supplementary material, movie S4). To identify Lgr5^+^-clusters, we tested the distribution of columnar epithelial cells using cytokeratin 8 staining. We found that Lgr5^+^-clusters were localized below the differentiated epithelial layer (figure 4*e*; electronic supplementary material, movie S5), and reconfirmed that differentiated epithelial cells spread on the surface of the gut (figures 4*e* and 3*d*). We further examined the movement of Lgr5-derived cells at the bottom of *ex vivo* gut (view from the bottom of petri dish; figure 5*a*). From day 1 to day 10, Lgr5-derived cells started to concentrate at two open ends of *ex vivo* guts, gradually spread into a large area and formed an epithelial layer on the surface of *ex vivo* guts (figure 5*a*,*b*). mRNA expression of *Foxl1* was further detected in *ex vivo* gut, suggesting Foxl1-expressing mesenchymal cells might facilitate the formation of stem cell niche [31] (electronic supplementary material, figure S3b).

### 3.4. Application of *ex vivo* culture to examine the role of AMPK in epithelial development

**Figure 6.**
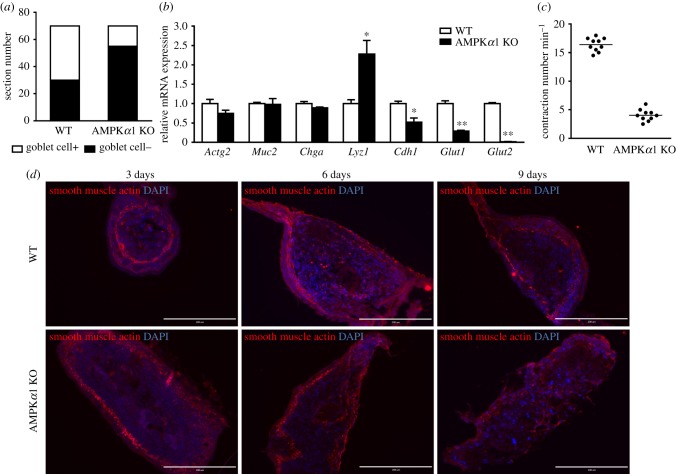
Differentiation of WT and AMPK*α*1 KO *ex vivo* guts. (*a*) Goblet cells positive *ex vivo* guts at 7 days. The *ex vivo* gut cross-sections were stained with Alcian blue, then goblet-positive and goblet-negative *ex vivo* guts were counted. A total of 70 *ex vivo* guts were used. (*b*) The mRNA expression of gut epithelial differentiation markers. (*c*) The contraction frequencies of *ex vivo* guts at 7 days were counted per minute. *n* = 10. (*d*) Immunostaining of smooth muscle actin for e*x vivo* guts at 3, 6 and 9 days to identify mesenchymal cells. DAPI was used as a nuclear stain. Scale bar is 200 µm. Data are represented as mean ± s.e.m. *p < 0.05; **p < 0.01.

The above established *ex vivo* culture system was further used to study the regulatory role of AMPK*α*1 on gut health from wild-type (WT) and AMPK*α*1 conditional knockout (AMPK*α*1 KO) mice. Alcian blue staining indicated that the prevalence of goblet cells was remarkably decreased in AMPK*α*1 KO *ex vivo* gut compared with that of WT (figure 6*a*). *Lyz1* was higher in AMPK KO group, while *Cdh1*, *Glut1* and *Glut2* were higher in WT group at mRNA level (figure 6*b*).

Under the normal physiological condition, guts undergo peristalsis. We found that WT *ex vivo* gut contracted and underwent peristalsis much stronger than that of AMPK KO at 7 days of culture (figure 6*c*; electronic supplementary material, movies S1 and S6). Because smooth muscle cells contribute to gut peristalsis, we further tested smooth muscle actin. In WT *ex vivo* guts, smooth muscle actin initiated to localize under the epithelial layer, and gradually spread along the contour of *ex vivo* gut; while in AMPK KO *ex vivo* guts, smooth muscle actin became increasingly disorganized over time (figure 6*d*).

**Figure 7.**
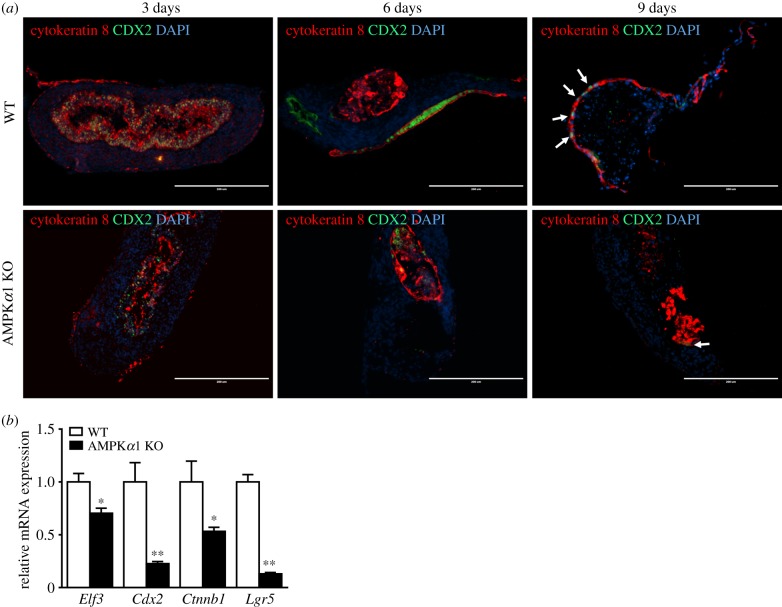
Expression of transcription factors and epithelial cells in WT and AMPK*α*1 KO *ex vivo* guts. (*a*) *Ex vivo* guts were cross-sectioned at 3, 6 and 9 days. The CDX2 transcription factor and epithelial cells were identified by immunostaining for Cytokeratin 8 and CDX2. Arrows point to CDX2-positive cells in the epithelial layer. DAPI was used as a nuclear stain. Scale bar is 200 μm. (*b*) mRNA levels of transcription factors in WT and AMPK KO *ex vivo* guts. Data are represented as mean ± s.e.m. *p < 0.05; **p < 0.01.

We further tested the effects of AMPK on epithelial cells of *ex vivo* guts. Both epithelial cells (cytokeratin 8 positive) and intestinal transcription factor (CDX2 positive) progressively moved from gut lumen to surface in WT *ex vivo* gut (figure 7*a*). However, this movement was absent in AMPK KO gut where both types of cells remained inside gut lumen (figure 7*a*), indicating that AMPK*α*1 deficiency unequivocally impeded the formation and overall arrangement of epithelial layers, which might be due to the downregulated mRNA expression of epithelial transcription factors (figure 7*b*).

**Figure 8.**
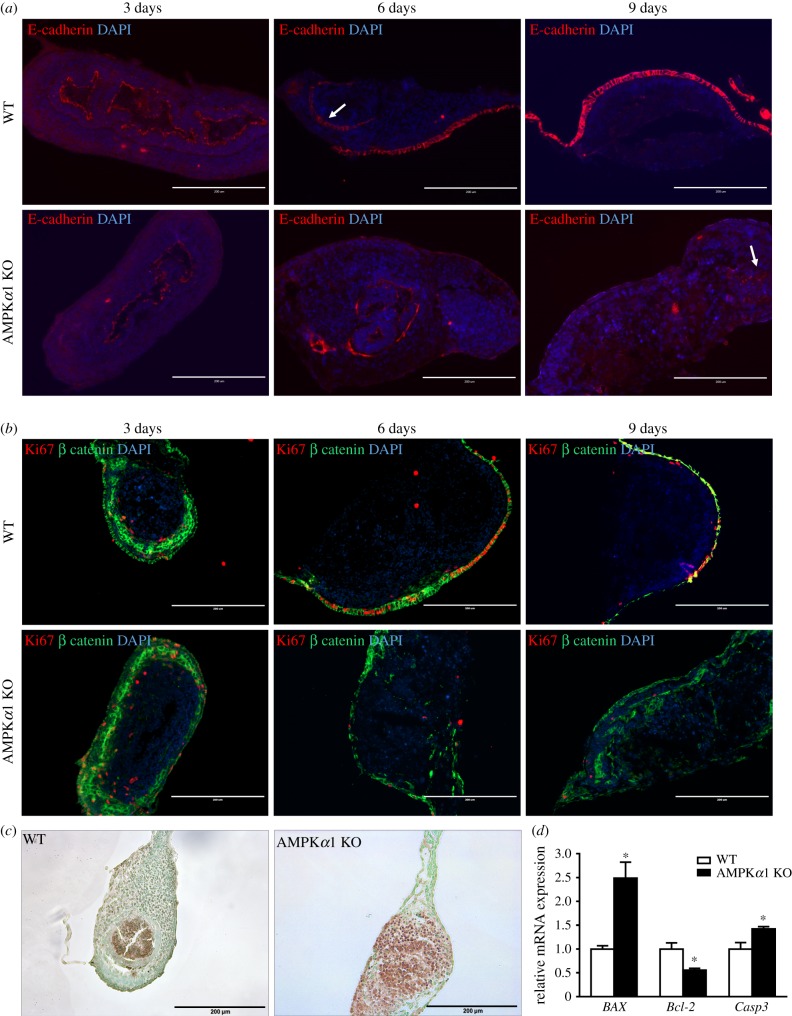
Epithelial junctions and proliferating and apoptotic cells of WT and AMPK*α*1 KO *ex vivo* guts. (*a*) *Ex vivo* guts were cross-sectioned at 3, 6 and 9 days. Junctions are determined by immunostaining for E-cadherin. Arrow points to the staining pattern in the lumen. (*b*) Adherens junction and proliferative cells were visualized by immunostaining for β-catenin and Ki67. DAPI was used as a nuclear stain. (*c*) Apoptotic cells of *ex vivo* guts at 7 days were visualized by TUNEL staining. Scale bar is 200 µm. (*d*) The mRNA expression of apoptotic markers of *ex vivo* guts at 7 days. Data are represented as mean ± s.e.m. *p < 0.05.

To further determine the role of AMPK in barrier function, E-cadherin and β-catenin, the markers for intestinal paracellular junction, were examined. Consistent with what we observed in figure 7*a*, E-cadherin and β-catenin in WT *ex vivo* guts gradually moved towards the exterior layer, and completely covered the surface layer over time; this phenomenon was absent in AMPK KO *ex vivo* guts (figure 8*a*,*b*). Since barrier function is also related to intestinal proliferation, we further tested Ki-67 (figure 8*b*). Ki67-positive cells were reduced due to AMPK*α*1 deficiency, which was consistent with the downregulated expression of *Ctnnb1* and *Lgr5* (figure 7*b*). Additionally, the apoptosis significantly induced after AMPK deficiency (figure 8*c*). Pro-apoptotic markers *BAX* and *Casp3* were upregulated in AMPK KO gut, while anti-apoptotic marker *Bcl-2* was downregulated (figure 8*d*). These data collectively demonstrated the necessity of AMPK*α*1 in proper gut health, and established *ex vivo* guts as a proper model for examining gut epithelial differentiation and formation.

## 4. Discussion

Epithelial cell lines and mice are commonly used models for studying epithelial health. An *in vivo* model is costly, time-consuming and often lacks specific niche for toxicological, pharmacological and nutraceutical studies. The recently developed organoid model from mouse intestinal stem cells [32] or from human-induced pluripotent stem cells [33] allows three-dimensional culture to form crypt-villus structures. These models use expensive growth factors and matrix gel embedding with low efficiency, and the functionality of smooth muscle is missing, making it inaccessible to study motility disorders [34].

The *ex vivo* gut culture system established in this study allows epithelium developing in a native gut niche without costly growth factors or other promoters. All steps of gut epithelial differentiation and migration can be closely monitored, which is difficult to do with live animals. Furthermore, inhibitors, activators, toxicants or carcinogens can be applied to *ex vivo* gut to study their biological effects on gut differentiation. This model was successfully applied to one nutraceutical study to determine the effects of purple potato extract on intestinal differentiation [35]. The shortcoming of the *ex vivo* gut culture is that gut sections are vulnerable to waggle and fall off from the coverslip during the first three days of culturing. Thus, careful handling is needed when moving culture dishes. Also, the defined crypt-villus structure could not be found in *ex vivo* guts. Thus, intestinal morphogenesis may not be applicable to this model.

During early gut development, the intestinal epithelial structure including crypts and villi starts to form around E14, a critical time window for intestinal development [36]. Thus, the embryonic intestine at E13.5 was used to establish the *ex vivo* gut culture model. During murine intestinal development, the major differentiated cells, such as absorptive enterocytes, goblet cells and enteroendocrine cells, can be detected [37], while Paneth cells can be detected from postnatal (P) day 14 [38]. CDX2, E-cadherin, smooth muscle actin, vimentin and chromogranin A were detected in the gut of E16.5 and E18.5 mice [33]. Alcian blue and Ki67 were also found in the epithelial layer of P4 mice duodenum [39]. In this study, similar staining in *ex vivo* guts has been detected. The difference between *ex vivo* model and *in vivo* is that *ex vivo* guts rearrange during culture, with epithelial cells distributing on the surface layer and mesenchymal cells concentrated under the epithelium as scaffolds.

Lgr5^mT/mG^ *ex vivo* gut was used to trace intestinal stem cells and their derived cells. Lgr5-derived intestinal cells ‘climb’ onto the surface of *ex vivo* gut, showing the migration of intestinal cells. On the other hand, intestinal stem cells give rise to enterocytes, goblet cells, enteroendocrine cells and Paneth cells, which were also discovered in *ex vivo* guts at 7 days post-incubation. Of note, Paneth cells, contributing to the niche of intestinal stem cells, normally start to differentiate from P14 in mice [38]. Paneth cells are detected in the organoid derived from a single intestinal stem cell after 14 days in culture [14]. In this study, Paneth cells were observed after 7 days in culture, which possibly differentiate to sustain epithelial stem cell niche [40].

The mesenchymal layers under the epithelial layer are the sources of growth factors and cytokines regulating epithelial differentiation [41,42]. There are several major cell types in the mesenchymal layers. Fibroblasts secrete collagen and synthesize connective tissue as the scaffold for epithelium, smooth muscle cells form contractile apparatus [43], and myofibroblasts possess both features [43]. In addition, smooth muscle cells cooperate with myofibroblasts to regulate contraction, while fibroblasts and myofibroblasts support gut structure. In *ex vivo* guts, the mesenchymal layers are clearly visible, which located below the epithelial layer; smooth muscle cells, fibroblasts and myofibroblasts were further detected. Importantly, the *ex vivo* gut demonstrates spontaneous peristalsis, very similar to the gut movement *in vivo*. Interstitial cells of Cajal (ICC), one type of interstitial cell in the gut, contribute to gut peristalsis, and the absence of ICC is related to motility disorders [30]. However, no ICC was detected, and peristalsis was even enhanced by neurotoxin in *ex vivo* guts, indicating that peristalsis in *ex vivo* gut is not due to ICC functionality. Likewise, botulinum toxin could even increase muscular activity in a spinal cord–muscle coculture system [44]. In short, our cultured *ex vivo* gut has mesenchymal layers which can be useful for studying epithelial motility.

To demonstrate the effectiveness of the model, we evaluated the role of AMPK*α*1 in embryonic gut epithelial development. We observed an astonishing difference between WT and AMPK*α*1 KO *ex vivo* gut. Our results are supported by recent reports showing the positive roles of AMPK in gut epithelial barrier function [25,45], as well as cell differentiation [25,46–48]. A previously published study in adult mice focused on AMPK loss in epithelial cells [25], while this study focused on AMPK loss in the whole guts including mesenchymal cells during development, suggesting the necessity of AMPK *α*1 in proper peristalsis and overall health of fetal gut. AMPK specific deficiency in intestinal epithelial cells promoted proliferation of crypt cells in adult mice [25], while AMPK deficiency in whole *ex vivo* gut suppressed the proliferation and induced apoptosis, possibly due to the subsequent apoptosis induced by inadequate or lagging differentiation. Consistently, AMPK mutants in *Drosophila* cause abnormal epithelial integrity, defective mitotic cell division and even embryonic lethality [49]. The deficient differentiation in AMPK*α*1 KO *ex vivo* gut is partially associated with the downregulation of intestinal transcription factors, such as CDX2 [25]. However, the stimulated proliferation in crypts of epithelial AMPK deficient gut could be due to the fully expressed AMPK in mesenchymal cells underneath crypts, which secretes growth factors and cytokines for tissue regeneration to activate crypt cell proliferation [50].

In summary, *ex vivo* gut culture system not only maintains the physiological structure of the gut but can effectively track intestinal epithelial differentiation and migration. Using the established *ex vivo* gut model, we further demonstrated a critical regulatory role of AMPK*α*1 in epithelial differentiation. This model has potential applications in examining impacts of nutrients, microbial metabolites, toxicants and antigenic materials on gut epithelial development, as well as exploring mechanisms regulating differentiation and migration.

## Supplementary Material

Supporting Information

## Supplementary Material

Figure S1

## Supplementary Material

Figure S2

## Supplementary Material

Figure S3

## Supplementary Material

Figure S4

## Data accessibility

This article has no additional data.
