# Supplementary material for: Ex vivo gut culture for studying differentiation and migration of small intestinal epithelial cells
Source: Open Biol. 2018 Apr 11;8(4):170256. doi: 10.1098/rsob.170256 (PMC5936714; doi:10.1098/rsob.170256)
Supplement: Supporting Information [file rsob170256supp1.docx]

**Supporting Information**

**Materials and Methods**

**Trypan blue staining**

Cell viability was tested using trypan blue staining as previously described [[1](#_ENREF_1)]. *Ex vivo* guts at 3, 5, 7 and 10 days were incubated with 0.067% trypan blue (Thermo Fisher Scientific, Waltham, MA) for 5 min, washed with PBS for 3 times, then left in PBS for 15 min at 37°C to remove unspecific staining. The images were taken using an inverted microscope (EVOS XL, Life Technology).

**Alkaline phosphatase activity**

Alkaline phosphatase assay was performed as previously described [[2](#_ENREF_2)]. *Ex vivo* guts collected at 0, 3, 5, 7 and 10 days were lysed and incubated with substrate and measured using a Synergy H1 microplate reader (BioTek, Winooski, VT) at 540 nm. 2 mm mouse tail from E13.5 embryos was used as a negative control.

**Neurotoxin treatment**

*Ex vivo* guts were cultured for 7 days and treated with 500 ng/ml botulinum neurotoxin (List Biological Laboratories, Campbell, CA) for 0, 6, 12 and 24 h. Each *ex vivo* gut from a dedicated mouse was considered as an experiment unit. The contraction frequencies of each set of *ex vivo* guts were counted per minute using an inverted microscope (EVOS XL, Life Technology). The relative contraction was calculated corresponding to the initial value at 0 h.

**Figure Legends**

**Figure S1. Cell viability, alkaline phosphatase activity and *Gata4* expression of *ex vivo* guts.** (a-f) Small intestines from E13.5 embryos were isolated and cultured to form *ex vivo* guts, and stained with trypan blue at 3 d (a), 5 d (b), 7 d (c), and 10 d (d). (e-f) Magnified image of a selected area in panel c-d. Scale bars are 400 μm in (a-d), and 200 μm in (e-f). (g) Alkaline phosphatase activity of *ex vivo* guts at 0 d, 3 d, 5 d, 7 d, and 10 d. Mouse tail from E13.5 embryos was used as a negative control (NC). Data are represented as mean ± s.e.m. n = 4. **: *p* < 0.01 versus 0 d. (h) Relative *Gata4* mRNA expression in e*x vivo* guts from embryonic distal or proximal small intestine after 7 days of culture. Data are represented as mean ± s.e.m. n = 4. *: *p* < 0.05 versus distal small intestine.

**Figure S2. Representative images of negative controls in immunofluorescent staining.** Sections were permeabilized, incubated with 5% goat serum overnight at 4℃, then with respective fluorescent secondary antibodies for 2 h at room temperature. Immunofluorescence images were taken using a fluorescence microscope. Scale bar is 200 μm.

**Figure S3. Interstitial cells of Cajal and Foxl1-expressing mesenchymal cells in *ex vivo* guts.** *Ex vivo* guts were cultured for 7 days. (a-b) Relative *C-kit* and *Foxl1* mRNA in *ex vivo* guts (EVG), intestine tissue of adult mice (positive control, PC) and fat tissue of adult mice (negative control, NC). (c) The relative contraction frequencies of *ex vivo* guts treated with botulinum neurotoxin for 0, 6, 12 and 24 h. Data are represented as mean ± s.e.m. Each *ex vivo* gut from a dedicated mouse was view as an experiment unit, n = 5. *: *p* < 0.05 versus 0 h. **: *p* < 0.01 versus 0 h.

**Figure S4. Effects of Wnt, BMP and Notch signaling on intestinal differentiation of *ex vivo* guts.** Small intestines from E13.5 embryos were isolated and cultured in medium containing 100 nM LDN-193189 (BMP signaling inhibitor; a), 10 μM DAPT (Notch signaling inhibitor; b), or 100 ng/ml Wnt 3a plus 500 ng/ml R-spondin1 (Wnt signaling activator; c). Relative mRNA expressions were analyzed using RT-qPCR. Data are represented as mean ± s.e.m. n = 4. *: *p* < 0.05 versus CON. **: *p* < 0.01 versus CON.

**Movie S1. Representative contraction of WT *ex vivo* guts.** The contraction frequencies of WT *ex vivo* guts at 7 d were counted per minute, which was presented in figure 6c. n =10.

**Movie S2. Representative Lgr5-derived epithelial cells in** ***ex vivo* guts at 3 days.** Lgr5^mT/mG^ *ex vivo* guts were induced by 250 μm 4-hydroxytamoxifen and the Lgr5-derived epithelial cells of *ex vivo* guts are shown at 3 days by confocal microscope. This movie is related to figure 4b.

**Movie S3. Representative Lgr5-derived epithelial cells in *ex vivo* guts at 6 days.** Lgr5^mT/mG^ *ex vivo* guts were induced by 250 μm 4-hydroxytamoxifen and the Lgr5-derived epithelial cells of *ex vivo* guts are shown at 6 days by confocal microscope. This movie is related to figure 4c.

**Movie S4. Representative Lgr5-derived epithelial cells in *ex vivo* guts at 9 days.** Lgr5^mT/mG^ *ex vivo* guts were induced by 250 μm 4-hydroxytamoxifen and the Lgr5-derived epithelial cells of *ex vivo* guts are shown at 9 days by confocal microscope. This movie is related to figure 4d.

**Movie S5. Representative Lgr5-derived epithelial cells and columnar epithelium in *ex vivo* guts at 7 days.** Lgr5^mT/mG^ *ex vivo* guts were induced by 250 μm 4-hydroxytamoxifen and the Lgr5-derived epithelial cells of *ex vivo* guts are shown at 7 days by confocal microscope. Columnar epithelium was stained with violet color. This movie is related to figure 4e.

**Movie S6. Representative contraction of AMPKα1 KO *ex vivo* guts.** The contraction frequencies of AMPKα1 KO *ex vivo* guts at 7 days were counted per minute, which was presented in figure 6c. n = 10.

**Table S1 Primers Information**

| **Gene** | **Pubmed ID** | **Size (bp)** | **Forward Primer** | **Reverse Primer** |
| --- | --- | --- | --- | --- |
| *Actg2* | NM_009610.2 | 155 | TGGGATGACATGGAGAAGATCTGG | GGGACATTGAAGGTTTCGAACATG |
| *Cdh1* | NM_009864.2 | 155 | GAAGCCATTGCCAAGTACATCCTCTAT | CCTTCTGCAACGAATCCCTCAAA |
| *Cdx2* | NM_007673.3 | 139 | CAGTCCCTAGGAAGCCAAGTGAAAA | CAGCCAGCTCACTTTTCCTCCTG |
| *Chga* | NM_007693.1 | 200 | GAGACCCTCCAAGGAGACGAGA | AGTTCATCCTCAAAGCTGCTGTGTT |
| *Ctnnb1* | NM_001165902.1 | 106 | TCAGTGCAGGAGGCCGA | CAGGTCAGCTTGAGTAGCCA |
| *Elf3* | XM_006529125.1 | 150 | GCAAGGTCTTCCCTAGAGATGGCT | GAGTACCTCTGGGGGCGTGCT |
| *Glut1* | NM_011400.3 | 152 | CAGCAGCAAGAAGGTGACGG | CTCCGTAGCGGTGGTTCCAT |
| *Glut2* | NM_031197.2 | 232 | GCTGCTGGATAAATTCGCCTG | CACGTAAGGCCCAAGGAAGT |
| *Muc2* | NM_023566.3 | 141 | ACGTCTGTGTGAAGACATGTGGATGT | CAGAACATTTCTTTGGTTGGCACA |
| *Lgr5* | NM_010195.2 | 185 | CACTCTCCAACCTTAAGGAACTAGGATTC | CGAGGCACCATTCAAAGTCAGTGT |
| *Lyz1* | NM_013590.4 | 137 | GTCAAGCTGGCTGACTGGGTGT | CCATCATTACACCAGTATCGGCTATTG |
| *Notch1* | NM_008714.3 | 210 | TGTTCGAGGACCAGATGGCTTCA | TTTGCAGCATCTGAACGAGAGTATCG |
| *Foxl1* | NM_008024.2 | 254 | GACAGCATCTTGGCCGTGAGA | GCTAATCCGCCTTAGTGGAAACG |
| *C-kit* | NM_001122733.1 | 225 | CGTTCCTCGCCTCCAAGAATTGTAT | CATAGGACCAGACATCACTTTCAAATGTG |
| *Gata4* | NM_001310610.1 | 336 | TGGAAGACACCCCAATCTCGTAGATA | ATTGCAAGAGGCCTGGGAACC |
| *18s* | 19791 | 110 | TGCTGTCCCTGTATGCCTCT | TGTAGCCACGCTCGGTCA |

**References**

[1] Pribluda, A., Elyada, E., Wiener, Z., Hamza, H., Goldstein, R. E., Biton, M., Burstain, I., Morgenstern, Y., Brachya, G., Billauer, H., A senescence-inflammatory switch from cancer-inhibitory to cancer-promoting mechanism. *Cancer cell* 2013, *24*, 242-256.

[2] Sun, X., Yang, Q., Rogers, C. J., Du, M., Zhu, M.-J., AMPK improves gut epithelial differentiation and barrier function via regulating Cdx2 expression. *Cell Death & Differentiation* 2017, *24*, 819-831.
