# Supplementary figures and images for: Ex vivo gut culture for studying differentiation and migration of small intestinal epithelial cells
Source: Open Biol. 2018 Apr 11;8(4):170256. doi: 10.1098/rsob.170256 (PMC5936714; doi:10.1098/rsob.170256)

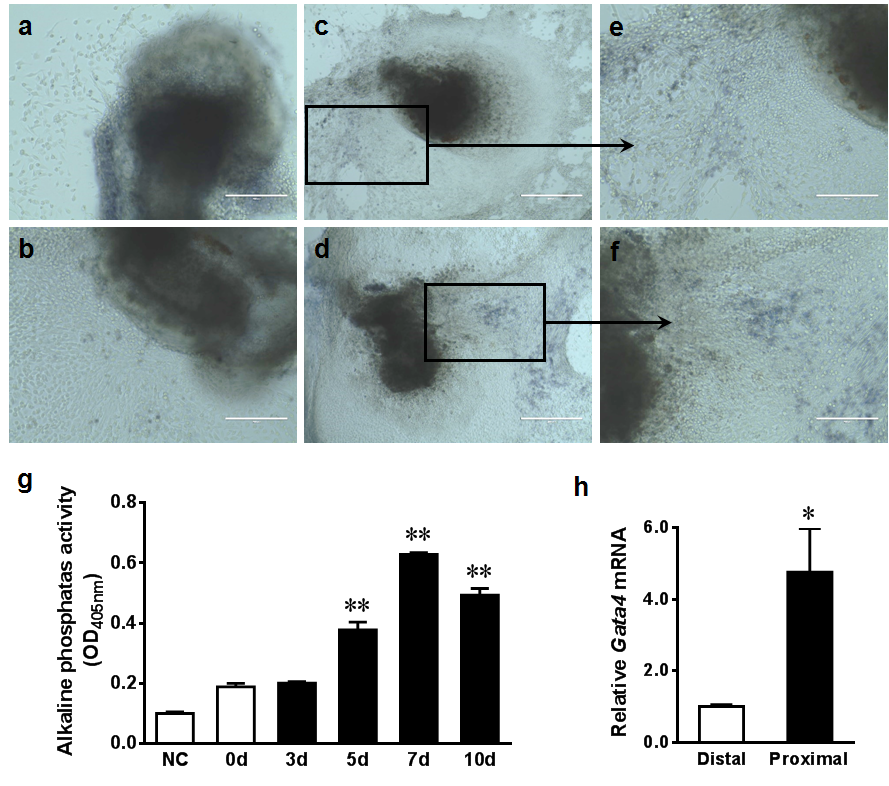

Supplement: Figure S1 [file rsob170256supp2.tif]

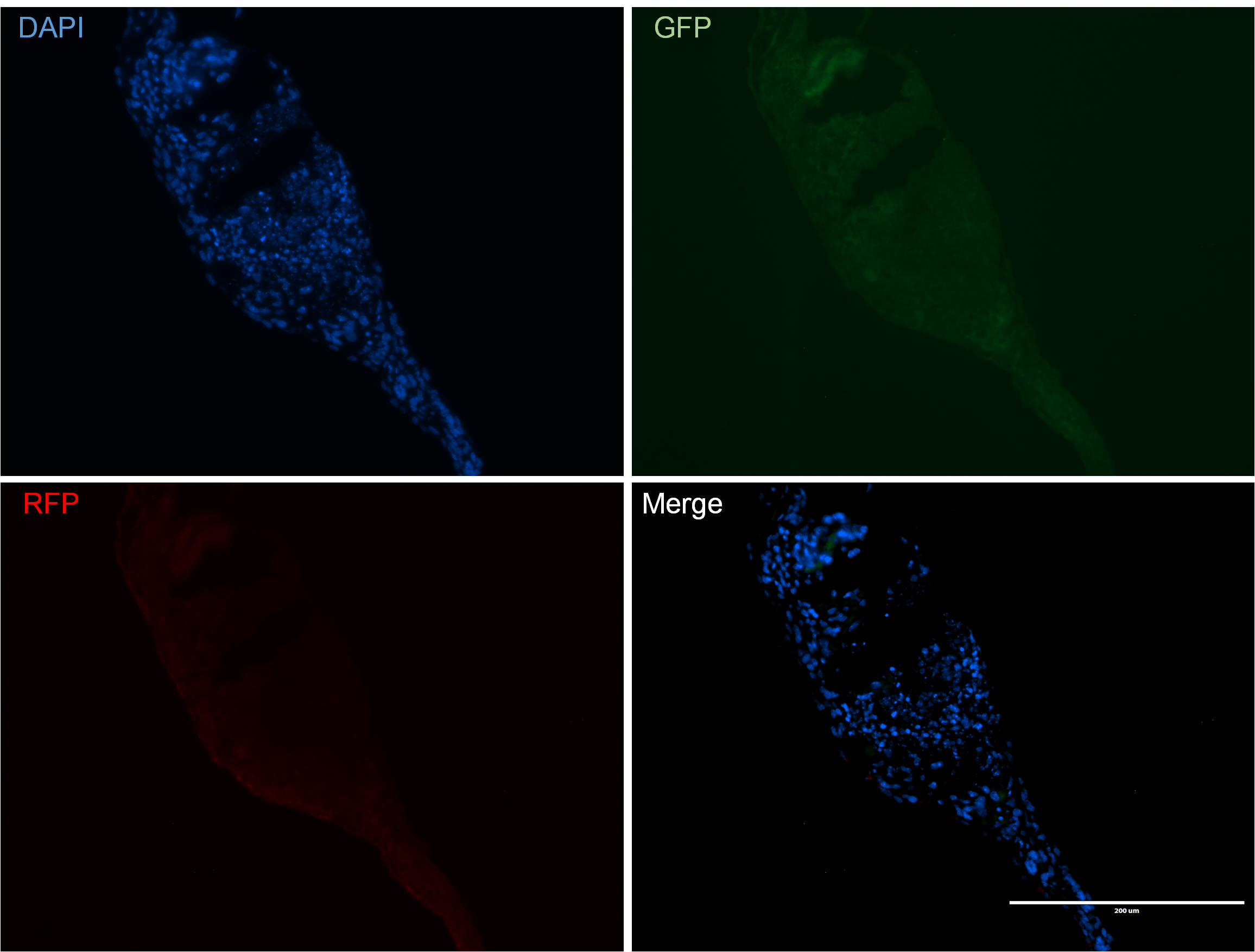

Supplement: Figure S2 [file rsob170256supp3.tif]

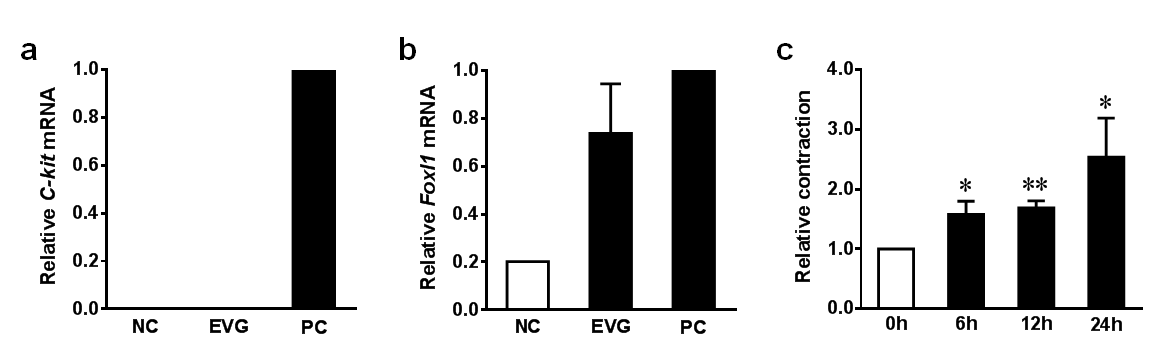

Supplement: Figure S3 [file rsob170256supp4.tif]

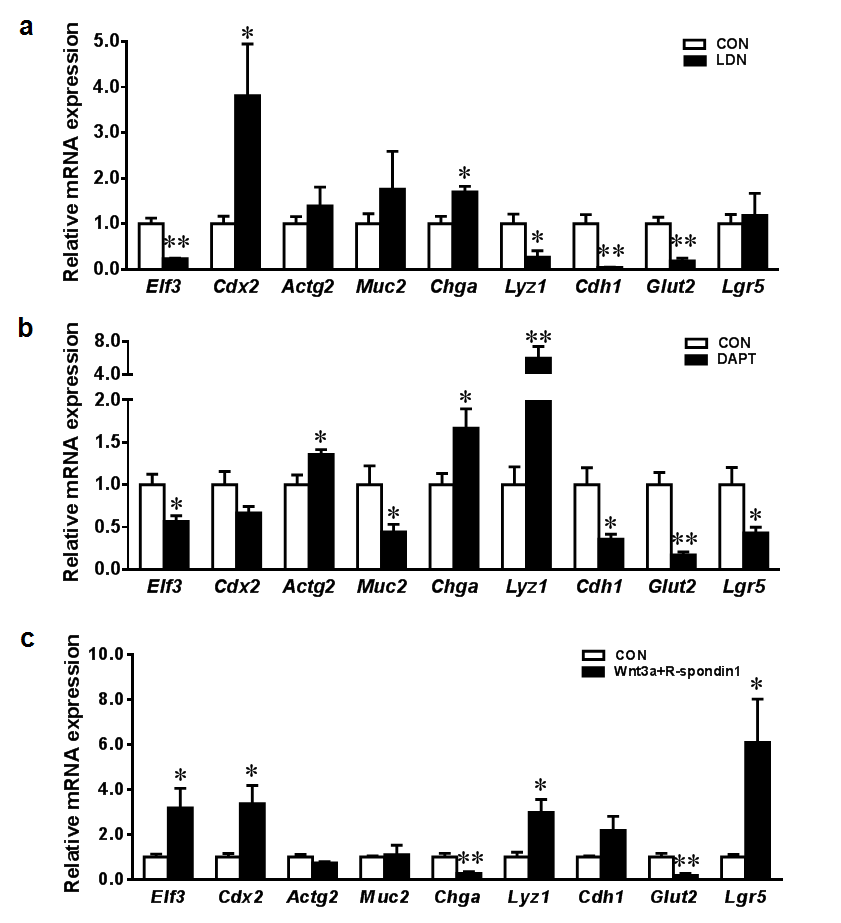

Supplement: Figure S4 [file rsob170256supp5.tif]
